# Seed Priming and Biopriming in Two Squash Landraces (*Cucurbita maxima* Duchesne) from Tunisia: A Sustainable Strategy to Promote Germination and Alleviate Salt Stress

**DOI:** 10.3390/plants13172464

**Published:** 2024-09-03

**Authors:** Néji Tarchoun, Wassim Saadaoui, Khawla Hamdi, Hanen Falleh, Ourania Pavli, Riadh Ksouri, Spyridon A. Petropoulos

**Affiliations:** 1 Research Laboratory LR21AGR05, High Agronomic Institute of Chott Mariem, Sousse University, Sousse 4042, Tunisia; nejitarchoun@yahoo.fr (N.T.); wessaadaoui@gmail.com (W.S.); hamdi.khawla21@gmail.com (K.H.); 2 Laboratory of Aromatic and Medicinal Plants, Center of Biotechnology, Technopark of Borj Cedria, BP 901, Hammam-Lif 2050, Tunisia; hanenfalleh@gmail.com (H.F.); ksouri.riadh@gmail.com (R.K.); 3 Laboratory of Genetics and Plant Breeding, Department of Agriculture, Crop Production and Rural Environment, University of Thessaly, Fytokou Street, 38446 Volos, Greece; ouraniapavli@uth.gr; 4 Laboratory of Vegetable Production, Department of Agriculture, Crop Production and Rural Environment, University of Thessaly, Fytokou Street, 38446 Volos, Greece

**Keywords:** seed germination, *Cucurbita maxima*, abiotic stress, salinity stress, hormopriming, bio-priming, *Trichoderma harzianum*, *Bacillus* sp.

## Abstract

In recent years, seed priming has gained interest, with researchers aiming to enhance seed germination and early growth, especially under abiotic stress conditions. In this study, seeds from two squash landraces (*Cucurbita maxima* Duchesne; i.e., Galaoui large seeds (Galaoui hereafter) and Batati green (Batati hereafter)) were subjected to different priming methods ((a) 0.3% and 0.4% KNO_3_ (halopriming); (b) 0.1% and 0.2% GA_3_ (hormopriming); (c) inoculation with *Trichoderma* spp. (*T. harzianum*, *T. viride*, and *T. virens*), *Bacillus subtilis*, and *Pseudomonas fluorescens* (biopriming) in order to promote germination parameters and seedling growth under salinity stress (0, 100, and 200 mM of NaCl). Our findings indicate the better performance of primed seeds compared to the untreated ones in terms of germination and seedling growth traits, although a varied response depending on the priming method and the landrace was observed. The highest germination percentage (GP) and the lowest mean germination time (MGT) were observed in 0.4% KNO_3_-primed seeds. The positive effects of 0.4% KNO_3_ were also depicted in all traits related to seedling growth and the seedling vigor index (SVI), indicating its effectiveness as a priming agent in squash seeds. Under salinity stress conditions, priming with 0.4% KNO_3_ significantly improved the germination and seedling growth traits for both landraces, while the application of 0.2% GA_3_ at high salinity significantly improved photosynthetic quantum yield (F_v_/F_m_ ratio). Regarding the effects of biopriming in germination and seedling growth traits, our results indicate that *T. harzianum* and *B. subtilis* were the most effective bioagents in promoting germination and seedling growth in Galaoui and Batati seeds, respectively. In conclusion, our findings provide important information regarding the practice of using priming and biopriming agents to enhance the germination and seedling growth capacity of squash seeds, as well to mitigate the negative effects of salinity stress at the critical stages of germination and early growth.

## 1. Introduction

In recent decades, changing climate conditions, involving global warming and aridity, along with poor-quality irrigation water and irrational fertilization management, have progressively led to increased soil salinization and agricultural land abandonment [1,2]. Salinity is considered to be one of the most common abiotic stresses that adversely affects plants throughout their growth cycle, leading to considerable yield losses, especially in arid and semi-arid regions [3,4]. Seed germination is considered the most sensitive stage for most plant species, and it determines crop establishment and subsequently crop performance [5]. Under salinity stress conditions, water absorption during seed imbibition and turgescence is limited [6,7], thus impairing seed germination and seedling growth due to osmotic, ionic, and oxidative stress [8,9,10]. These conditions may induce complex interactions at the morphological, physiological, biochemical, and molecular level that ultimately lead to germination inhibition and seedling death [11,12].

The effects of salinity stress during germination are manifested through reduced seed germination percentage and germination rate and increased germination time [13,14,15,16]. The response of salt-sensitive species to such conditions includes a series of changes in physiological processes, such as photosynthetic apparatus and the accumulation of reactive oxygen species (ROS) [4,17]. Chlorophyll fluorescence is routinely employed as a tool with which to assess plant tolerance to environmental stressors and determine their effects on the photosynthetic apparatus [18] since the ROS-induced damage of chloroplasts leads to reduced chlorophyll content and to the inhibition of photosynthesis [19]. Moreover, the response of plants to salinity stress involves the activation of antioxidant enzymes and the accumulation of osmolytes, which are responsible for cell membrane stability and protein structure [20,21].

Apart from stress conditions (e.g., stress duration and severity) and plant traits, sensitivity to salinity stress may be affected by the longevity of seeds, which is influenced by genetic factors as well as the pre-storage and storage conditions, mainly associated with the initial seed moisture content and the relative humidity and temperature under storage [22]. Based on their behavior under storage, seeds are classified as orthodox, intermediate. and recalcitrant, while it has been well documented that conventional storage conditions are not appropriate for short-lived and desiccation-sensitive seeds, as is the case of most horticultural crops whose seeds are characterized as orthodox seeds [23].

In recent years, seed priming has gained ground as an alternative, low-cost approach to enhancing the germination potential of orthodox seeds, as well as to mitigating abiotic stress effects in various plant species [24,25]. In this context, various chemical and physical methods have been suggested, including hydropriming, potassium nitrate, gibberellic acid-priming (GA_3_), cytokinin, chlorhydric acid, sodium chloride, thioredoxin, sulphuric acid, seed scraping, etc. [11,25]. Seed priming has been proven to be a valuable tool with which to enhance, accelerate, and synchronize the germination of seeds, thus leading to improved seedling and crop establishment while breaking seed dormancy and preventing seed deterioration under unfavorable conditions for seed germination [24]. More importantly, recent scientific evidence suggests that seed priming induces tolerance to stressors, such as drought and salinity, thus improving yield potential in diverse ecosystems [26,27,28]. The beneficial effects of seed priming have been reported in various crops, including summer squash (priming with GA_3_, KNO_3_, and polyethylene glycol (PEG)) [27,29], pepper (biopriming with *Trichoderma harzianum*, *T. viride*, *Pseudomonas fluorescens*, *Bacillus subtillis*, *Paecilomyces lilacinus*, and hydropriming) [30], green bean ((biopriming with *T. harzianum*, *T. viride*, *P. fluorescens*, and hydropriming) [31], tomato (priming with GA_3_, Napthlene acetic acid (NAA), and potassium nitrate (KNO_3_)) [32], and fennel (priming with priming with GA_3_, KNO_3_, and hydropriming) [33].

Seed priming involves a series of biochemical, physiological, and cellular responses that occur from seed imbibition and germination initiation up to radicle emergence. Seed germination involves a triphasic kinetic of water uptake, initialized by a rapid uptake (imbibition phase), followed by a lag phase (activation phase), and it is completed with the elongation of the embryonic axis (germination phase) [34]. Priming accelerates the second phase, increases the number of germinating seeds, synchronizes the radicle emergence, and enhances the seedling growth rate [34]. Furthermore, seed priming induces the hydrolysis of germination inhibitors and the activation of enzymes—mainly α-amylase and dehydrogenase—that hydrolyze the starch reserves into smaller sugars, providing energy to the developing embryo [24]. In general, seed priming is defined as a pre-sowing treatment, using various agents that mediate the partial hydration of seeds without allowing germination, as well as dehydration to the original moisture content [35]. The most commonly used priming treatments are hydropriming, osmopriming, halopriming, and hormopriming, referring to the use of water, osmotic agents, salts, and plant growth regulators, respectively [36]. In addition, apart from physical and chemical seed priming techniques, biopriming has emerged as a promising approach towards enhancing the germination potential of seeds, particularly of organic ones through the use of bioagents [25]. In this context, several studies underline the beneficial effect of bioagents, such as arbuscular mycorrhizal fungi, *Trichoderma* spp., rhizobia, and plant-growth-promoting bacteria (*Pseudomonas* spp., *Bacillus* spp.) in seed germination of cereal and vegetable crops [37,38,39].

In Tunisia, squash (*C. maxima* Duch.) germplasm used for commercial cultivation, both in conventional and organic farming, is essentially derived from local landraces obtained via the open pollination or farmer mass selection and maintained by local farmers [40]. Nevertheless, the seeds of local landraces often exhibit decreased viability and germination ability, mainly as a result of inappropriate storage conditions (high temperatures and relative humidity) and extended storage period [40]. The loss of seed viability during storage reflects the onset of deterioration processes, involving irreversible cellular damage, which is manifested through delayed seedling emergence, reduced tolerance to abiotic stresses, and consequently, loss of viability [41]. *C. maxima* is moderately sensitive to salt stress [29]; however, the response of the species varies considerably depending on stress intensity and duration but also on factors related to genotype characteristics [8,42]. Despite the recent reports related to the beneficial effects of priming and biopriming approaches, there is a relevant gap for data addressing the interactive effect of various priming methods and salinity stress on seed germination traits of squash. Therefore, the main objectives of this study were to assess the following: (1) the effects of various priming (halopriming and hormopriming) treatments in traits related to squash germination and seedling growth in seeds grown with conventional cropping practices; (2) the effects of biopriming treatments in traits related to squash germination and seedling growth in seeds grown with organic cropping practices; and (3) the potential of using priming techniques as an eco-friendly and sustainable tool with which to mitigate the adverse effects of salinity stress on seed germination of two local squash landraces.

## 2. Results

### 2.1. Effect of Seed Priming on Germination and Seedling Growth

The results of the ANOVA regarding the effects of seed priming on the germination and seedling growth of the two studied squash landraces are presented in Table 1. The obtained data revealed a significant effect of the priming treatments (*p* < 0.001) on the germination traits (DP and MGT) and seedling growth (RL, SL, SL/RL ratio, and SVI) of the two squash landraces. Germination potential and seedling vigor index under different priming treatments were differentially affected by landraces, which could be primarily attributed to the seed size of those landraces. A significant interaction between landrace × priming was recorded for GP, SVI, MGT, and SL (Table 1).

**Figure 1:**
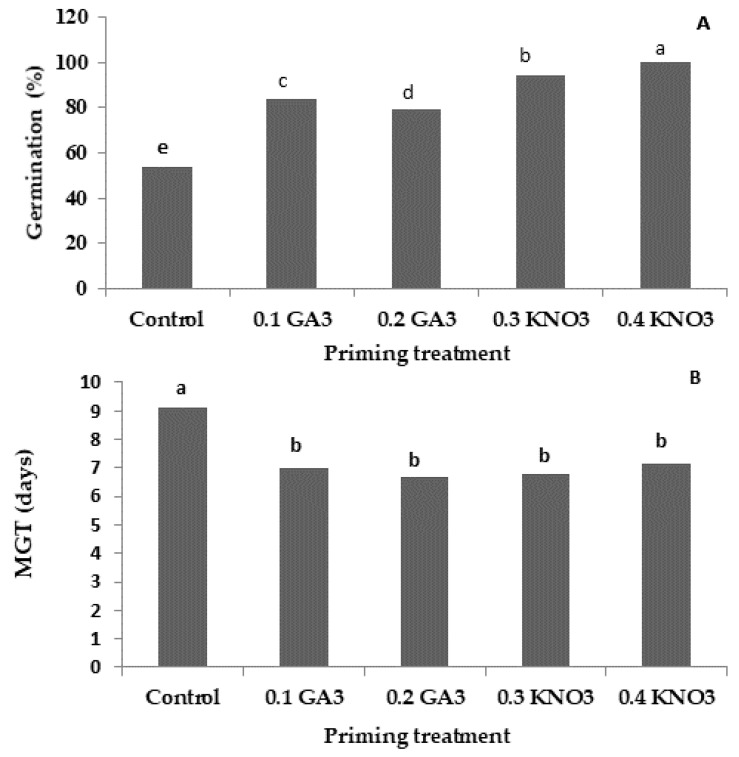
The effect of priming treatments on the germination percentage (GP) (**A**) and mean germination time (GMT) (**B**) of the studied squash landraces. Different Latin letters above the bars indicate significant differences according to Duncan’s multiple range test (DMRT) at *p* < 0.05.

Germination percentage (GP) varied significantly (*p* < 0.001) among the priming treatments, ranging from 69.15% to 100%. The highest GP was recorded in primed seeds with 0.4% of KNO_3_ (100%), followed by 0.3% of KNO_3_ (94.28%), whereas the lowest values were noted in non-primed seeds (53.90%) (Figure 1A). Regarding the mean germination time (MGT), an average time gain of 2 days compared with the non-primed seeds was recorded (Figure 1B). Such findings are indicative of the positive effects of seed priming on both GP and MGT. Moreover, the analysis revealed the significant effect of the priming agent, as well as its concentration on GP, as evidenced by the significant differences among the studied priming treatments, whereas no differences in MGT values were recorded.

Regarding the seedling growth traits, RL, SL, SL/RL ratio, and SVI differed significantly among different priming treatments (Table 2). For RL, values ranged from 3.48 to 5.35 cm, while the lowest and highest values were recorded for non-primed (control) and seeds primed with 0.4% KNO_3_, respectively. Accordingly, SL ranged from 5.18 to 6.78 cm, where the most- and least- effective treatments were the application 0.4% KNO_3_ and 0.1% GA_3_, respectively. In relation to the above-mentioned values, the lowest SL/RL ratio was recorded in seeds primed with 0.2% and 0.1% GA_3_ (1.11 and 1.12, respectively), whereas the highest ratio was recorded in non-primed seeds due to their lowest root length values (1.52), although no significant differences were recorded from the treatment of 0.3% KNO_3_. Finally, the SVI values differed significantly among the priming treatments, with the lowest and highest values being recorded in the seeds treated with 0.2% GA_3_ and 0.4% KNO_3_, respectively. Our data suggest the superior performance of primed seeds in relation to seedling growth traits, yet they present variations subjected both to the type and concentration of the priming agent.

### 2.2. Effect of Landrace on Germination and Seedling Growth

Our findings suggest the significant effect of landrace on germination percentage and SVI, a trait also related to germination, while landraces did not differ in terms of seedling growth traits and mean germination time (Table 3). In particular, the Galaoui landrace showed higher values for both GP and SVI (87.88% and 902.52, respectively), as compared to the Batati landrace, thus indicating either a better response to the priming treatments applied or a higher innate germination ability.

### 2.3. Effect of Seed Priming under Saline Conditions

In non-primed seeds, the increasing salt stress level considerably affected all traits related to germination and seedling growth in both squash landraces (Table 4). Salinity stress significantly compromised the germination potential, leading to a severe decrease in the final germination percentage compared to controls (no NaCL added) 14 days after stress initiation (Galaoui: 21% and 48.6%; Batati: 5% and 48% at 100 mM and 200 mM NaCl, respectively), as well as a delay in germination, as evidenced by the MGT values (Table 4). Further, the effects of salt stress were also manifested on seedling growth, with the most profound effects being noted at 200 mM NaCl in both roots and shoots length. At 200 mM NaCl, the decrease over the control treatment was 43% and 36% for RL and SL for Galaoui seeds, respectively; while for Batati, the respective values were 29% and 22%. Consequently, the SL/RL ratio presented an increasing trend as the stress was intensified in both landraces, reaching an 84% and 21% increase over the control treatment at 200 mM NaCl in Galaoui and Batati, respectively, thus indicating the increased sensitivity of roots as compared to the shoots (Table 4).

**Figure 2:**
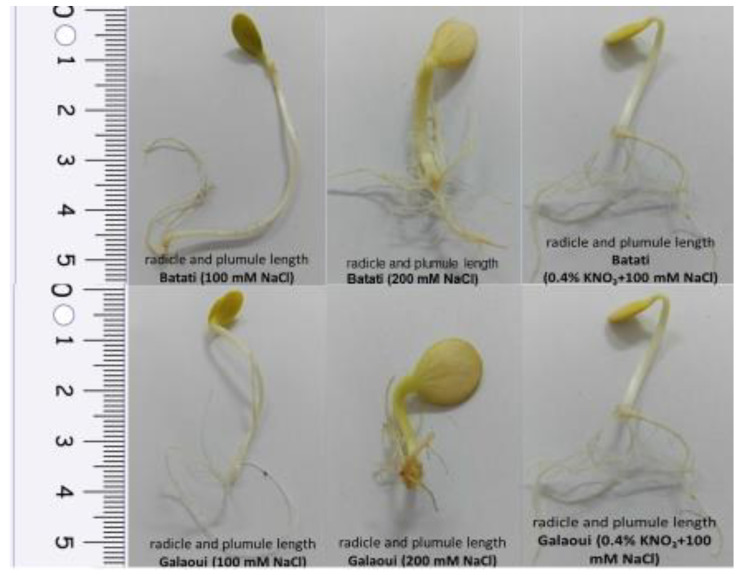
Radicle and plumule length in non-primed seeds of Galaoui and Batati squash landraces subjected to NaCl-induced salinity stress (100 mM and 200 mM NaCl) and in 0.4% KNO_3_-primed seeds subjected to 100 mM NaCl-induced salt stress.

Moreover, our results indicate the positive effects of all priming treatments on traits related to germination and seedling growth (Table 4). At moderate salinity stress (e.g., 100 mM NaCl), GP values ranged from 89.2 to 100.0% and 71.7 to 87.8% in Galaoui and Batati seeds, respectively, suggesting a significant increase both over the control and the respective treatment, where no NaCl was added. It is worth mentioning that priming with 0.4% KNO_3_ pronouncedly enhanced the germination potential of both landraces, especially for Galaoui seeds, where all seeds germinated, while the application of 0.1% GA_3_ resulted in higher GP values compared to 0.2% GA_3_ for both landraces (Figure 2). Similarly, GMT values were considerably decreased when seems were subjected to priming, especially for Galaoui seeds (5.31 to 6.31 days), whereas the effect was less evident in Batati seeds (7.76 to 8.59 days). On the other hand, priming alleviated the negative effects of salinity stress on seedling growth, as depicted in the RL and SL values, especially in the case of Batati seeds, where values were higher than the control and the 100 mM NaCl treatments.

At 200 mM of NaCl, both landraces showed a significant reduction in growth parameter values compared to the control treatment, while priming resulted in higher GP, RL, and SL and lower MGT and SL/RL values compared to the 200 mM NaCl treatment for both landraces (Table 4). A varied response was recorded for the tested priming agents depending on the landrace, with seeds of Galaoui showing a better response to KNO_3_ application in terms of SL and RL, while the same priming agent resulted in significantly increase GP values compared to the non-primed seeds subjected to 200 mM NaCl. Moreover, both priming agents reduced MGT by 29.1 to 33.5% and 8.8 to 18.5% in Galaoui and Batati seeds, respectively.

In order to investigate the potential of using priming treatments as a means to mitigate salinity stress effects further, we evaluated chlorophyll fluorescence as well as the content of Chla, Chlb, and carotenoids (Table 5). The highest overall values for pigments content were recorded for the non-stressed seedlings treated with 0.3% KNO_3_, whereas the lowest ones for the non-primed seeds subjected to severe salinity stress (200 mM NaCl). On the other hand, chlorophyll fluorescence was the highest for seedlings treated with 0.2% GA_3_ and subjected to 200 mM NaCl, whereas the lowest values were recorded under moderate salinity (100 mM NaCl) and for the application of 0.1% GA_3_.

For the non-stressed seedlings, most of the priming treatments improved the studied traits, except for 0.1% GA_3_, which differed significantly compared to the control treatment (non-primed seedlings), while no significant differences were recorded for the F_v_/F_m_ ratio between the non-primed and primed seedlings. Moreover, the application of KNO_3_ significantly improved the chlorophyll and carotenoids content, regardless of the applied concentration (e.g., 0.3% or 0.4%).

Under salinity stress conditions, non-primed seedlings showed a decreased content of Chl a, Chl b, and carotenoids compared to the primed seedlings for both landraces (Table 5). In particular, the lowest overall values for the content of photosynthetic pigments were recorded in non-primed seeds subjected to 200 mM NaCl-induced salinity stress (8.73, 3.27, and 0.59 mg/g FW for Chla, Chlb, and carotenoid content, respectively). Moreover, under saline conditions and for both salinity levels, the application of 0.4% KNO_3_ resulted in the highest content of pigments, thus indicating the efficiency of using this priming agent as a stress mitigation tool.

### 2.4. Effect of Seed Bio-Priming on Germination and Seedling Growth

The effect of different bioagents on seed germination and seedling growth was investigated in two landraces, i.e., Batati and Galaoui, and the results are presented in Table 6. Our results revealed that all the tested bioagents were capable of improving the traits related to germination and seedling growth in both landraces, although a varied response between the genotypes was recorded. In particular, GP was improved in all the primed seeds, with significant differences noted among the tested bioagents and landraces, while the lowest GP values were recorded for the control treatment (non-primed seeds). On the other hand, the highest over values were recorded in seeds primed with *T. harzianum* and *B. subtilis* for Galaoui (91.65%) and Batati (81.12%) landraces, respectively, while Galaoui seeds recorded higher CP values than the Batati ones, regardless of the priming treatment. The positive effects of bio-priming were further evidenced in MGT, which was significantly reduced by all the priming treatments compared to the untreated seeds, which recorded the highest MGT values. The highest acceleration of seed germination was noted in seeds treated with *T. harzianum* and *T. virens* for Galaoui (5.12 days) and Batati (4.76 days), respectively. In contrast, the longest germination time was recorded in non-primed seeds for both Galaoui (8.68 days) and Batati (8.32 days).

Moreover, the tested bioagents were effective in promoting seedling growth in both landraces through the increase in root and shoot length compared to the control treatment, which recorded the lowest values. Among the bioagents, *T. harzianum* proved to be the most effective in enhancing RL in both Galaoui and Batati (10.27 and 10.01 cm, respectively), while *T. virens* also increased rot length in Galaoui seedlings (10.14 cm). Similarly, *T. virens* and *T. harzianum* were the most effective bioagents in enhancing SL in the Galaoui (12.78 and 12.58 cm, respectively) landrace, while the shoot length of the Batati seedlings was mostly benefited by *B. subtilis* (11.63 cm). Finally, all the tested bioagents resulted in higher SVI values compared to the control treatment, which recorded the lowest overall values for both landraces (Table 6). The most effective bio-agent was *T. harzianum*, which resulted in the highest SVI values for both landraces (988.31 and 800.17 for Batati and Galaoui landraces, respectively), whereas the lowest SVI values were recorded for *B. subtilis* and *P. fluorescens* in Galaoui (730.41) and Batati (544.00), respectively.

## 3. Discussion

Our results indicate that priming of seeds resulted in improved germination in both landraces, as evidenced by the increased GP and the decreased MGT compared to the non-primed (control) seeds. Among priming treatments, 0.4% KNO_3_ was the most efficient for both landraces, leading to a mean increase in GP by 85% and a mean decrease in MGT of 2.4 days. Moreover, the seedlings obtained from seeds primed with 0.4% KNO_3_ exhibited an enhanced growth potential, as indicated by the 29% and 54% increase over the control treatment for shoot and root length, respectively, as well as by the SVI values, which were also drastically increased by 75%. The positive effects of priming with KNO_3_, expressed by improved seed germination, seed dormancy breaking [43], and seedling growth uniformity, have been reported in various species, including tomato [44], pea [45], and rice [46]. However, the exact underlying mechanisms of action have not been yet well elucidated. According to the literature, it has been proposed that KNO_3_ modulates ABA metabolism or ABA signaling in developing seeds since the activation of ABA catabolism and GAs biosynthesis is required for seed germination [47]. Moreover, Moaaz Ali et al. [48] suggested that low NO^3−^ concentration decreases ABA content, leading to the induction of the *CYP707A2* gene, which encodes an ABA 8′-hydroxylase involved in ABA catabolism. In the same line, it is well documented that gibberellins play a crucial role in numerous physiological plant processes, including seed germination [49]. In our study, the seeds primed with 0.1% GA_3_ showed an increased GP by 55% as well as a decreased MGT of 2.1 days compared to the non-primed seeds. In accordance with our findings, several studies reported the positive impact of halopriming with KNO_3_, as well as of hormopriming with GA_3_ on seed germination and seedling growth, both under normal and stress conditions in various plant species, including soybean (seed priming with 6 g L^−1^ of KNO_3_) [49], tomato (seed priming with 0.25, 0.50, 075, 1.0, and 1.25 KNO_3_ (*w*/*v*)) [48], and rice (seed priming with GA3 at 5, 10, 50, and 100 mg L^−1^, among other priming agents) [50]. Therefore, the positive results of our study and those of the literature reports could be directly exploited to enhance the value of seeds, especially those that are self-produced by farmers and usually subjected to limiting factors such as long-term storage and seed dormancy, which contribute to poor or erratic germination.

Addressing the performance of primed seeds under salt stress conditions, our findings underline that seed priming facilitates the mitigation of salinity stress on seed germination and seedling growth. More specifically, seed priming with 0.4% KNO_3_ alleviated the effects of 100 mM NaCl-induced salinity stress, as manifested by the increased GP values of up to 27% and 7.6% over the control treatment for Galaoui and Batati seeds, respectively. On the other hand, priming with 0.1% GA_3_ resulted in increased GP values by up to 19% and 3.3% compared to the untreated seeds of Galaoui and Batati landraces, respectively. Reports in the literature have suggested that the underlying mechanism of action for GA_3_ priming is associated with the expression of genes that are responsible for the α-amylase mRNA transcription, which reduces starch into sucrose and glucose into the newly germinating embryo, thus providing the required energy for seed germination, as well as with the increased activity of lipase and protease enzymes, which lead to improved seed vigor and germination index [51,52].

It is worth noting that the observed varied response of Galaoui and Batati seeds to the tested priming agents could be related to differences in seed size, as it is well documented that the progress of seed water uptake during germination may vary depending on the seed size, thus affecting germination and seedling development in various plant species, such as wheat [53] and leafy vegetables [54]. However, seed size varies widely among different crop species and growth environments, especially in local landraces and populations where a significant variability in various traits is exhibited. In general, large seeds present higher seedling growth, an improved survival rate, and better field performance than small seeds under non-stressful environments [55]. Moreover, varietal differences in response to priming could be attributed to differences in the content of seeds in starch and other energy reserves, which greatly affects germination and early seedling growth [56,57].

In our study, seeds subjected to high salinity stress (e.g., 200 mM NaCl) were severely affected in terms of GP and the overall germination process in both landraces. In particular, GP was reduced in both landraces by up to 48%, while a considerable increase was also noted in MGT (up to 26%) and SL/RL ratio (up to 84%), the latter indicating the higher sensitivity of roots than shoots under salinity conditions. These findings are consistent with the reported effects of salinity stress levels on the seed germination of various plant species, including lentil [58], tomato, and soybean [59], among others. Moreover, considering that roots are responsible for water absorption and shoots for supplying aboveground tissues with water, root and shoot length are the most suitable traits by which to evaluate salinity tolerance, since they are greatly affected under saline conditions [60,61]. The high sensitivity of seeds to salinity during the germination stage is associated with a series of physiological, biochemical, and molecular adjustments resulting from the combined effect of osmotic and ionic stress, oxidative stress, and water absorption imbalance, which ultimately leads to germination inhibition [8,10,11].

Seed bio-priming has emerged as a low-cost and eco-friendly technique that may enhance seed germination potential, especially in the organic farming sector. This approach refers to pre-sowing treatment of seeds with beneficial microorganisms, especially those with low longevity [39]. In our study, *Trichoderma* strains and other bacterial agents, namely, *P. fluorescens* and *B. subtilis,* were investigated for their potential to promote germination and seedling growth in the organic seeds of Galaoui and Batati landraces. Although all the tested bioagents were effective in increasing the seed germination percentage of both landraces compared to the non-primed seeds, our findings suggest a varied response of landraces to the studied bioagents, thus suggesting that the bioproming technique should be optimized based on the genotype. Such findings are further supportive of previous evidence related to the beneficial effects of bio-priming on germination and seedling growth traits in several plant species, including wheat [62], soybean and maize [63], and cucumber [64]. In the same line, several reports suggested that seed coating with arbuscular mycorrhizal fungi, capable of producing gibberellins, may induce primary root emergence as well as the elongation of lateral roots in several species [65,66], thus improving the early growth of seedlings and providing protection to biotic and abiotic stressors [66,67].

Recently, the application of plant-growth-promoting microbes or other biostimulants in seed bio-priming has been suggested as a novel approach by which to boost seed germination, improve seedling vigor, and enable uniform seedling emergence effectively [68,69]. Moreover, bio-priming with arbuscular mycorrhizal fungi, such as *Trichoderma* spp., or certain bacterial strains may mitigate the negative effects of abiotic stressors on critical germination both during and after radicle emergence [64]. Such effects are associated with the establishment of early symbiotic relationships with plants, thus affecting nutrient and water uptake and contributing to improved resilience to abiotic stresses [70]. In our study, *Trichoderma* spp. has been proven to be the most efficient bio-agent in enhancing seedling growth, especially in the Galaoui landrace, where an increase by 54% and 100% was recorded in shoot and root length, respectively. On the other hand, the Batati landrace mostly benefited from priming with *T. harzianum* and *B. subtilis*, which increased root and shoot length, respectively. Several studies support that such effects of bio-priming agents on root tissues may reflect the result of cellular changes related to root morphology, cell wall composition, and the accumulation of secondary metabolites and phytohormones, which positively affect root elongation and ramification processes [71,72]. In this context, it has been reported that *B. pumilus* INR-7 and *P. fluorescens* 63–28R promote lignin deposition in the root cell wall of *Pennisetum glaucum* L. and *Pisum sativum* L. [73], while also promoting the assimilation of nutrients, such as NO^3−^ and K^+^, and accelerating their cellular transport through root cells by enhancing the activity of proton pumps [73]. Moreover, cell wall modifications in roots have been associated with the induction of systemic resistance through several signaling pathways [74].

## 4. Materials and Methods

### 4.1. Plant Material

Two local Tunisian squash landraces (*Cucurbita maxima*) were selected from a pool of fifteen squash landraces based on the data related to their response to salt stress, published by our team [40], in order to evaluate their response to different priming agents further, either in the absence or the presence of NaCl-induced salinity stress. The selected landraces represent the main types of early- and late-cultivated squash in Tunisia, namely, Batati green (“NGB1008”) and Galaoui large seeds (“NGB1004”), respectively. Each landrace was assigned passport data and an inventory number, according to the National Gene Bank of Tunisia (NGB), while full details are available at the Germplasm Resources Information Network-GRIN (accessed on 1 July 2024; http://www.tn-grin.nat.tn/gringlobal/search.aspx). Landrace seeds were stored in a cold room (± 4 °C and 50% Relative humidity (RH)) for a period of 3 years at the NGB. The evaluation of the response to biopriming agents was performed using the seeds of the two landraces, which were propagated by the Technical Center of Organic Agriculture (CTAB; latitude 35°54′22.21″ N and longitude 10°32′47.81″ E) Chott Mariem-Sousse and traditionally stored over a number of last years.

### 4.2. Seed Priming Treatments

The priming experiments were carried out at the Vegetable Laboratory, Department of Horticulture, High Agronomic Institute of Chott Mariem, Tunisia (latitude 35°54′22.21″ N and longitude 10°32′47.81″ E). The first experiment was conducted to determine the effect of different seed priming treatments on the germination percentage and the seedling characteristics of Batati and Galaoui squash landraces. The seed priming treatments included the following: (i) hormonal, using gibberellic acid (GA_3_) at 0.1% and 0.2% (*w*/*v*, diluted in distilled water (H_2_O)); and (ii) halopriming, using potassium nitrate (KNO_3_) at 0.3% and 0.4% (*w*/*v*, diluted in dH_2_O). Non-primed seeds (e.g., not treated prior to sowing) were used as the control treatment.

The required number of seeds (200 seeds per landrace in 4 replications) were selected for size homogeneity, and seeds were subsequently surface-sterilized using 1% hypochlorite/H_2_O solution under gentle agitation for 5 min and washed 4 times with the excess of the sterile distilled H_2_O. Pre-sowing treatments involved the soaking of the sterilized seeds in their respective solutions (or with distilled water in the case of non-treated seeds) and incubating them for 24 h at room temperature, according to the method described by Kamra et al. [27]. Then, seeds were air-dried until they reached the original moisture level (approximately 10%) via incubation at room temperature for 4–5 h. For seed germination assays, seeds were placed on sterile glass petri dishes (9 cm of diameter) containing two layers of filter paper and incubated under controlled conditions (25 ± 2 °C, 50 ± 5% relative humidity, and 18 h light/6 h dark photoperiod), according to the ISTA [75]. Seeds were moistened daily with 5 mL of their respective solutions (or with distilled water in the case of non-treated seeds), and seed germination was recorded daily for a period of 14 days.

### 4.3. Combination of Seed Priming and Salt Stress Treatments

The second experiment aimed to assess the potential of seed priming in alleviating the effects of salt stress during the germination and early-growth stages. Therefore, a combination of priming agents (0.1% and 0.2% GA_3_, 0.3%, and 0.4% KNO_3_) and salt stress levels (100 and 200 mM NaCl) was applied. In total, ten treatments were applied (T2–T11) and compared to the control, as described in Table 7. Two hundred seeds per landrace in four replications were evaluated.

For pre-sowing treatments, seeds were soaked in 5 mL of their respective solutions (or in distilled water for the control treatment) and incubated for 24 h at room temperature conditions. Seeds were subsequently sown in 104-cell honeycomb plastic trays (200 cm^3^) that were filled with commercial peat. The experiment was performed in three replicates, each consisting of fifteen seeds for each landrace × treatment combination. Trays were kept in a glasshouse for a period of two months (1 February–30 March 2022; mean air temperature: 21/12 °C ± 2 °C (day/night); relative humidity: 65–70%).

### 4.4. Seed Bio-Priming Treatments of Organic Seeds

The third experiment was conducted to determine the effect of bio-priming agents on the germination percentage and seedling characteristics of Batati and Galaoui landraces seeds obtained via organic cultivation. Initially, the required number of organic seeds (200 seeds per landrace in 4 replications) were selected for size homogeneity and surface-sterilized, as mentioned above (Section 2.1). Following seed drying to the original moisture level, seeds were soaked for 4 h in different *Trichoderma* spp. strains (10^6^ cfu mL^−1^) and bacterial bioagents (*Pseudomonas* and *Bacillus*) (10^8^ cfu mL^−1^) that were supplied by ISA-CM’s Phytopathology and Bacteriology Laboratory.

### 4.5. Evaluation Criteria

For all the experiments, the effects of priming agents under study were assessed on the basis of traits related to seed germination and seedling growth. In particular, germination percentage (GP), mean germination time (MGT), root and shoot length (RL, SL, respectively), and seedling vigor index (SVI) were used as the evaluation criteria. In addition, in the second experiment, where the objective was to assess the potential of seed priming in alleviating the effects of salt stress, the evaluation criteria also included physiological parameters, which are routinely employed as screening criteria for abiotic stress tolerance. More specifically, the chlorophyll fluorescence [18] and the content of chlorophyll a and b and carotenoids were determined in mature leaves of squash plants [76].

#### 4.5.1. Evaluation of Seed Priming Effects on Seed Germination and Seedling Growth

The seed priming effects were evaluated on the basis of various traits related to seed germination and seedling growth potential, according to the following formula:

Germination percentage $\left(GP\right)=\frac{number\;germinated\;seeds}{number\;of\;total\;seeds}\times$ 100 [77]. Germination percentage was scored daily for a period of 14 days until no germinated seeds were further recorded. Seeds were considered as germinated when the radicle had emerged from the seed coat and had a length of at least 2 mm.

Mean germination time (MGT) was measured using the equation described by Ellis and Roberts [78]: MGT $=\frac{\sum \left(Dn\right)}{\sum \left(n\right)},$ where *D* is the number of days and *n* is the number of seeds germinated on day *D*.

For the seedling vigor index, we used the following formula: (SVI) = (Root length + Shoot length) × GP, in cm [79].

Root and shoot length (RL, SL) were measured using a thread and scale.

The ratio of shoot length to root length (SL/RL) was estimated using the respective values for shoots and roots.

#### 4.5.2. Evaluation of Seed Priming Effects under Salinity Stress Conditions

The performance of primed seeds under salinity stress conditions was evaluated on the basis of traits related to seed germination and seedling growth, as well as physiological parameters, which serve as screening criteria for salt tolerance. Seedling emergence was recorded daily upon appearance of the hypocotyls above the substrate surface and expressed as a percentage by dividing the number of emerged seedlings by the total number of seeds sown at 14 days after sowing. Shoot and root length were determined in 15 seedlings for each landrace × treatment combination, which were transplanted in 2 L pots containing peat and soil and transferred to a greenhouse. Physiological traits, namely, chlorophyll fluorescence and content of chlorophyll a and b and carotenoids, were evaluated at 45 days after sowing, when the plants had reached the stage of having 4 true leaves.

##### Estimation of Chlorophyll Fluorescence

Chlorophyll fluorescence in the leaves of seedlings was measured on the 3rd fully expanded healthy leaf using a fluorometer (Plant Stress Kit, Opti-Sciences model, Hudson, NH, USA) based on the methodology of Maxwell and Johnson [18]. The dark adaptation period and the level of saturating light were determined before measurements. The values of the maximum quantum yield (F_v_/F_m_ quantum ratio; F_v_: variable fluorescence; F_m_: maximum fluorescence) were determined for each landrace × treatment combination.

##### Determination of Chlorophyll a,b and Carotenoids Content

Chlorophyll a and b were determined according to the method of Porcar-Castell et al. [76]. Briefly, 0.1 g of leaf tissue was ground in 10 mL of 80% acetone. Following filtration, the extracts were incubated in the dark to avoid photo-oxidation. The content of chlorophyll a (Chla) and chlorophyll b (Chlb) was measured using a UV/VIS spectrophotometer (Evolution 210, Thermo Scientific, Abingdon, UK) at 645 and 663 nm, respectively, whereas carotenoids (Car) content was determined at 470 nm [80]. The calibration of the apparatus was performed using 80% acetone. The relationship between concentration (mg/g FW) and optical density was determined according to the following formulae described by Arnon [81]:

Chla (mg/g) = 12.7 × D_663_ − 2.59 × D_645_,

Chlb (mg/g) = 22.9 × D_645_ − 4.68 × D_663_,

Car (mg/g) = [(5 × D_470_) × (3.19 × Chla)) + (130.3 × Chlb)]/200,

where D is the optical density value at the respective wavelengths.

### 4.6. Statistical Analysis

All the experiments were performed using a completely random design (CRD) with four replications (n = 4). Data were subjected to two-way analysis of variance (ANOVA). Means were compared using the Duncan’s multiple range test (DMRT) at *p* < 0.05. Statistical analyses were performed using the SAS software V9.2 (SAS Institute, Cary, CA, USA).

## 5. Conclusions

Our work suggested that both chemical and biological processes were effective in increasing seed germination parameters (e.g., germination percentage and mean germination time) and seedling growth as expressed by root and shoot length and the seed vigor index in two Tunisian landraces of squash subjected to salinity stress and non-stress conditions. In particular, KNO_3_ was effective in alleviating salinity stress effects on the seeds and seedlings of both landraces, while the same priming agent showed promising results under control conditions (no salinity stress). In the same line, the tested bioagents increased germination and seedling growth parameters in a genotype-dependent manner. In conclusion, seed priming should be considered as an eco-friendly and sustainable tool with which to improve crop establishment and increase the crop performance of cultivated species, especially under unfavorable conditions (e.g., salinity stress). However, further research is needed to optimize the priming protocols in accordance with the particularities of each species.

## Figures and Tables

**Table 1:** Analysis of variance (mean squares) results for traits related to the seed germination and seedling growth of two squash landraces under different priming treatments (control, 0.1% GA_3_, 0.2% GA_3_, 0.3% KNO_3_, and 0.4% KNO_3_).

S.O.V.	DF	GP	MGT	RL	SL	SL/RL	SVI
Landrace	1	974.02 **	0.05 ^ns^	0.39 ^ns^	0.07 ^ns^	0.03 ^ns^	59108.88 **
Priming	4	1911.83 **	6.01 **	3.37 **	3.85 **	0.20 **	480674.83 **
Landrace × Priming	4	82.12 **	1.49 *	0.68 ^ns^	0.97 *	0.03 ^ns^	52746.42 **
CV (%)	-	3.98	9.51	11.52	8.86	10.60	8.32

S.O.V.: source of variance; CV: coefficient of variance; DF: degrees of freedom; GP: germination percentage; RL: root length; SL: shoot length; SL/RL: shoot-length-to-root-length ratio; MGT: mean germination time; SVI: seedling vigor index. ^ns^: non-significant; *: significant at *p* < 0.1; **: significant at *p* < 0.01.

**Table 2:** Mean effect of priming treatment on traits related to seedling growth of squash landraces.

Treatment	RL (cm)	SL (cm)	SL/RL Ratio	SVI
Control	3.48 ^b^	5.26 ^b^	1.52 ^a^	692.38 ^d^
0.1% GA_3_	4.68 ^a^	5.18 ^b^	1.12 ^c^	810.66 ^c^
0.2% GA_3_	5.26 ^a^	5.79 ^b^	1.11 ^c^	509.05 ^e^
0.3% KNO_3_	4.85 ^a^	6.84 ^a^	1.42 ^ab^	1065.90 ^b^
0.4% KNO_3_	5.35 ^a^	6.78 ^a^	1.27 ^bc^	1212.67 ^a^
F. value	5.20 **	6.70 **	4.76 **	39.18 **

RL: root length; SL: shoot length; SL/RL: shoot-length-to-root-length ratio; SVI: seedling vigor index. Values with the same letter in each column are similar according to Duncan’s multiple range test (DMRΤ) at *p* ≤ 0.05. **: significant at *p* < 0.01.

**Table 3:** Mean effect of two squash landrace on traits related to germination and seedling growth.

Landrace	GP (%)	MGT (Days)	RL (cm)	SL (cm)	SL/RL Ratio	SVI
Galaoui	87.88 ^a^	7.30 ^a^	4.61 ^a^	5.92 ^a^	1.31 ^a^	902.52 ^a^
Batati	76.49 ^b^	7.38 ^a^	4.84 ^a^	6.02 ^a^	1.25 ^a^	813.74 ^b^
F value	76.11 **	5.87 ^ns^	5.20 ^ns^	6.70 ^ns^	4.76 ^ns^	39.18 **

RL: root length; SL: shoot length; SL/RL: shoot-length-to-root-length ratio; SVI: seedling vigor index. Values with the same letter in each column are similar according to Duncan’s multiple range test (DMRΤ) at *p* ≤ 0.05. ^ns^: non-significant; **: significant at *p* < 0.01.

**Table 4:** The response of two squash landraces to different priming treatments in terms of germination and seedling growth potential under NaCl-induced salinity stress.

Landrace	Treatment	GP (%)	MGT (days)	RL (cm)	SL (cm)	SL/RL
Galaoui	Control	78.66 ± 2.17 ^c^	7.61 ± 1.05 ^ab^	5.82 ± 0.65 ^a^	6.87 ± 1.04 ^a^	1.05 ± 0.41 ^d^
	100 mM NaCl	62.22 ± 2.63 ^d^	8.56 ± 2.36 ^ab^	4.35 ± 0.25 ^bc^	5.08 ± 0.59 ^bc^	1.43 ± 0.20 ^b^
	200 mM NaCl	40.44 ± 4.63 ^e^	9.61 ± 3.07 ^a^	3.33 ± 0.54 ^c^	4.39 ± 0.56 ^d^	1.93 ± 0.22 ^a^
	100 mM + 0.1% GA_3_	94.00 ± 5.12 ^b^	6.31 ± 1.37 ^b^	4.22 ± 1.02 ^bc^	5.56 ± 1.23 ^bc^	1.14 ± 0.42 ^de^
	100 mM + 0.2% GA_3_	90.00 ± 4.69 ^b^	6.10 ± 1.06 ^bc^	4.68 ± 1.00 ^bc^	5.74 ± 1.20 ^ab^	1.83 ± 0.44 ^b^
	100 mM + 0.3%KNO_3_	89.20 ± 5.58 ^b^	5.97 ± 1.64 ^bc^	5.32 ± 1.12 ^ab^	5.55 ± 0.98 ^bc^	1.37 ± 0.25 ^c^
	100 mM + 0.4%KNO_3_	100.00 ± 0.00 ^a^	5.31 ± 2.05 ^bc^	5.38 ± 1.15 ^ab^	5.89 ± 1.05 ^b^	1.56 ± 0.19 ^b^
	200 mM + 0.1% GA_3_	66.11 ± 2.93 ^d^	6.61 ± 3.01 ^ab^	3.39 ± 0.89 ^c^	4.81 ± 0.87 ^c^	1.43 ± 0.27 ^bc^
	200 mM + 0.2% GA_3_	62.22 ± 2.63 ^d^	6.81 ± 1.96 ^ab^	3.41 ± 0.53 ^c^	4.52 ± 0.48 ^c^	1.92 ± 0.15 ^a^
	200 mM + 0.3%KNO_3_	63.88 ± 3.33 ^d^	6.39 ± 1.17 ^bc^	4.35 ± 0.34 ^bc^	5.33 ± 1.25 ^bc^	1.80 ± 0.30 ^ab^
	200 mM + 0.4%KNO_3_	68.11 ± 4.85 ^d^	6.75 ± 2.09 ^bc^	4.89 ± 0.84 ^ab^	5.63 ± 1.22 ^b^	1.50 ± 0.21 ^b^
Batati	Control	81.55 ± 2.20 ^ab^	8.58 ± 2.59 ^b^	4.35 ± 1.05 ^b^	5.28 ± 1.54 ^c^	1.4 ± 0.10 ^b^
	100 mM NaCl	77.22 ± 2.63 ^b^	8.33 ± 3.17 ^bc^	3.89± 0.56 ^c^	4.66 ± 1.27 ^c^	1.2 ± 0.20 ^b^
	200 mM NaCl	42.22 ± 1.63 ^ef^	9.75 ± 2.89 ^a^	3.10 ± 0.84 ^cd^	4.12 ± 0.98 ^d^	1.7 ± 0.11 ^a^
	100 mM + 0.1% GA_3_	84.25 ± 5.78 ^a^	8.59 ± 2.29 ^b^	4.15 ± 1.02 ^c^	4.65 ± 0.85 ^c^	1.7 ± 0.20 ^a^
	100 mM + 0.2% GA_3_	71.66 ± 2.50 ^c^	7.76 ± 2.37 ^c^	3.95 ± 0.57 ^bc^	4.37 ± 1.07 ^c^	1.3 ± 0.10 ^b^
	100 mM + 0.3%KNO_3_	82.77 ± 2.83 ^ab^	7.89 ± 2.92 ^c^	4.88 ± 1.04 ^ab^	5.27 ± 1.24 ^bc^	1.6 ± 0.12 ^ab^
	100 mM + 0.4%KNO_3_	87.77 ± 2.88 ^a^	8.13± 3.15 ^bc^	4.89 ± 1.12 ^ab^	5.66 ± 1.42 ^b^	1.15 ± 0.30 ^bc^
	200 mM + 0.1% GA_3_	45.00 ± 1.73 ^e^	8.89 ± 3.17 ^ab^	4.39 ± 1.52 ^b^	5.89 ± 0.95 ^a^	1.16 ± 0.10 ^bc^
	200 mM + 0.2% GA_3_	45.00 ± 1.73 ^e^	8.75 ± 2.37 ^ab^	4.91 ± 1.45 ^a^	5.78 ± 1.24 ^b^	1.26 ± 0.11 ^b^
	200 mM + 0.3%KNO_3_	62.22 ± 2.44 ^d^	8.23 ± 2.14 ^bc^	3.91 ± 0.45 ^d^	4.97 ± 1.32 ^bc^	1.33 ± 0.30 ^b^
	200 mM + 0.4%KNO_3_	63.00 ± 2.48 ^d^	7.95 ± 2.38 ^c^	4.23 ± 0.86 ^b^	5.86 ± 0.94 ^ab^	1.35 ± 0.50 ^b^

Data are shown as means ± SD. GP: germination percentage; MGT: mean germination time; RL: root length; SL: shoot length; SL/RL: shoot-length-to-root-length ratio; SVI: seedling vigor index. Values with the same letter in each column and for each landrace combination treatment are similar according to Duncan’s multiple range test (DMRΤ) at *p* ≤ 0.05.

**Table 5:** Mean effect of priming treatment on the content of Chl a, Chl b (mg mg^−1^), carotenoids (mg mg^−1^), and chlorophyll fluorescence (F_v_/F_m_ ratio) of two squash landraces under NaCl-induced salt stress conditions.

Treatment	Chl a	Chl b	Carotenoids	F_v_/F_m_
Control	13.02 ^d^	5.10 ^abc^	0.83 ^de^	0.80 ^cd^
0.1% GA_3_	12.93 ^d^	4.42 ^cd^	0.76 ^g^	0.80 ^cd^
0.2% GA_3_	14.38 ^b^	5.77 ^abc^	0.86 ^c^	0.80 ^cd^
0.3% KNO_3_	14.55 ^a^	6.47 ^a^	0.95 ^a^	0.81 ^c^
0.4% KNO_3_	14.18 ^c^	6.37 ^ab^	0.85 ^cd^	0.81 ^c^
100 mM NaCl	11.11 ^h^	4.55 ^bcd^	0.76 ^g^	0.81 ^c^
200 mM NaCl	8.73 ^j^	3.27 ^d^	0.59 ^i^	0.80 ^cd^
100 mM NaCl + 0.1% GA_3_	11.12 ^h^	4.28 ^cd^	0.75 ^g^	0.77 ^f^
100 mM NaCl + 0.2% GA_3_	11.96 ^e^	4.85 ^bcd^	0.82 ^e^	0.81 ^c^
100 mM NaCl + 0.3% KNO_3_	11.69 ^f^	4.53 ^bcd^	0.80 ^f^	0.83 ^b^
100 mM NaCl + 0.4% KNO_3_	12.97 ^d^	5.44 ^abc^	0.89 ^b^	0.81 ^c^
200 mM NaCl + 0.1% GA_3_	8.85 ^j^	3.38 ^d^	0.65 ^h^	0.80 ^cd^
200 mM NaCl + 0.2% GA_3_	9.73 ^i^	6.10 ^abc^	0.66 ^h^	0.84 ^a^
200 mM NaCl + 0.3% KNO_3_	9.64 ^i^	4.22 ^cd^	0.64 ^h^	0.77 ^f^
200 mM NaCl + 0.4% KNO_3_	11.44 ^g^	4.59 ^bcd^	0.78 ^fg^	0.83 ^b^

Data are shown as means ± SD. Values with the same letter in each column are similar according to Duncan’s multiple range test (DMRΤ) at *p* ≤ 0.05.

**Table 6:** The effect of bio-priming agents on traits related to germination and seedling growth of two squash landraces.

Bio-Agent	Squash Landrace	GP (%)	MGT (Days)	RL (cm)	SL (cm)	SVI
Control	Galaoui	65.45 ± 0.81 ^cd^	8.68 ± 0.06 ^a^	5.13 ± 0.68 ^d^	8.16 ± 0.16 ^c^	568.92 ± 1.51 ^c^
	Batati	63.58 ± 1.05 ^cd^	8.32 ± 0.08 ^a^	5.01 ± 0.45 ^d^	7.82 ± 0.28 ^c^	291.26 ± 0.87 ^d^
*T. harzianum*	Galaoui	91.65 ± 1.87 ^a^	5.61 ± 0.08 ^b^	10.27 ± 0.49 ^a^	12.58 ± 0.26 ^a^	988.31 ± 0.51 ^a^
	Batati	73.45 ± 0.95 ^c^	5.12± 0.10 ^b^	10.01 ± 0.58 ^a^	10.24 ± 0.25 ^bc^	800.17 ± 1.01 ^ab^
*T. viride*	Galaoui	89.75 ± 1.54 ^a^	7.40 ± 0.02 ^a^	8.75 ± 0.61 ^b^	11.09 ± 0.22 ^ab^	898.45 ± 0.88 ^a^
	Batati	75.42 ± 1.14 ^bc^	6.27 ± 0.09 ^a^	8.35 ± 0.77 ^bc^	10.46 ± 0.21 ^b^	767.80 ± 1.05 ^b^
*T. virens*	Galaoui	84.87 ± 1.15 ^ab^	6.17 ± 0.10 ^a^	10.14 ± 0.51 ^a^	12.78 ± 0.18 ^a^	875.18 ± 1.21 ^ab^
	Batati	78.18 ± 0.85 ^bc^	4.76 ± 0.03 ^c^	8.37± 0.64 ^c^	10.33 ± 0.31 ^bc^	715.63 ± 1.37 ^b^
*P. fluorescens*	Galaoui	88.71 ± 1.01 ^ab^	7.15 ± 0.06 ^a^	8.41 ± 0.66 ^b^	10.43 ± 0.28 ^bc^	740.27 ± 1.54 ^b^
	Batati	70.14 ± 0.82 ^c^	7.78 ± 0.04 ^a^	9.11 ± 0.74 ^ab^	10.57 ± 0.34 ^b^	544.00 ± 1.00 ^c^
*B. subtilis*	Galaoui	89.19 ± 0.94 ^b^	6.15 ± 0.02 ^ab^	8.76 ± 0.66 ^ab^	11.75 ± 0.27 ^ab^	730.41 ± 1.16 ^b^
	Batati	81.12 ± 0.98 ^b^	8.11 ± 0.09 ^a^	9.11 ± 0.72 ^ab^	11.63 ± 0.23 ^ab^	684.25 ± 1.22 ^bc^

Data are shown as means ± SD (*n* = 50). GP: germination percentage; MGT: mean germination time; RL: root length; SL: shoot length; SL/RL: shoot-length-to-root-length ratio; SVI: seedling vigor index. Values with the same letter in each column are similar according to Duncan’s multiple range test (DMRΤ) at *p* ≤ 0.05.

**Table 7:** Combination of pre-sowing seed priming and salinity treatments.

Treatment	Composition	Treatment	Composition
T1	Control	T7	0.4% KNO_3_ + 100 mM NaCl
T2	100 mM NaCl	T8	0.1% GA_3_ + 200 mM NaCl
T3	200 mM NaCl	T9	0.2% GA_3_ + 200 mM NaCl
T4	0.1% GA_3_ + 100 mM NaCl	T10	0.3% KNO_3_ + 200 mM NaCl
T5	0.2% GA_3_ + 100 mM NaCl	T11	0.4% KNO_3_ + 200 mM NaCl
T6	0.3% KNO_3_ + 100 mM NaCl		

## Data Availability

Data are available upon request.
